# Possible role of mitochondrial K-ATP channel and nitric oxide in protection of the neonatal rat heart

**DOI:** 10.1007/s11010-018-3370-4

**Published:** 2018-05-25

**Authors:** Jan Doul, Dana Miková, Marcela Rašková, Ivana Ošťádalová, Hana Maxová, Bohuslav Ošťádal, Zuzana Charvátová

**Affiliations:** 1 Department of Pathophysiology, Second Faculty of Medicine, Charles University, Plzeňská 130/221, 150 06 Prague 5, Czech Republic; 2 Department of Physiology, Second Faculty of Medicine, Charles University, Prague, Czech Republic; 3 Institute of Physiology, The Czech Academy of Sciences, Prague, Czech Republic

**Keywords:** Neonatal rats, Ischemic postconditioning, Tolerance to ischemia, Mito-K-ATP channel, Nitrates, 3-Nitrotyrosine

## Abstract

Cardioprotective effect of ischemic preconditioning (IPC) and ischemic postconditioning (IPoC) in adult hearts is mediated by mitochondrial-K-ATP channels and nitric oxide (NO). During early developmental period, rat hearts exhibit higher resistance to ischemia–reperfusion (I/R) injury and their resistance cannot be further increased by IPC or IPoC. Therefore, we have speculated, whether mechanisms responsible for high resistance of neonatal heart may be similar to those of IPC and IPoC. To test this hypothesis, rat hearts isolated on days 1, 4, 7, and 10 of postnatal life were perfused according to Langendorff. Developed force (DF) of contraction was measured. Hearts were exposed to 40 min of global ischemia followed by reperfusion up to the maximum recovery of DF. IPoC was induced by 5 cycles of 10-s ischemia. Mito-K-ATP blocker (5-HD) was administered 5 min before ischemia and during first 20 min of reperfusion. Another group of hearts was isolated for biochemical analysis of 3-nitrotyrosine, and serum samples were taken to measure nitrate levels. Tolerance to ischemia did not change from day 1 to day 4 but decreased on days 7 and 10. 5-HD had no effect either on neonatal resistance to I/R injury or on cardioprotective effect of IPoC on day 10. Significant difference was found in serum nitrate levels between days 1 and 10 but not in tissue 3-nitrotyrosine content. It can be concluded that while there appears to be significant difference of NO production, mito-K-ATP and ROS probably do not play role in the high neonatal resistance to I/R injury.

## Introduction

Cardiac ischemia is the leading cause of morbidity and mortality in developed countries. It originates as a result of disproportion between myocardial oxygen supply and demand. The degree of ischemic injury depends not only on the intensity and duration of ischemic stimuli but also on the level of cardiac tolerance to oxygen deprivation. This particular parameter changes significantly during ontogenetic development: we have recently shown that the time course in rats shows a biphasic pattern [1]. The relatively high cardiac resistance at birth increases up to the end of the weaning period; then declines in males but remains unchanged in females; the adult female heart is thus significantly more resistant to oxygen deprivation [2].

Cardiac tolerance to ischemia in adult animals can be significantly increased by different protective phenomena, such as adaptation to chronic hypoxia and ischemic pre- and postconditioning (IPC, IPoC). We have shown previously that the above protective interventions were not able to increase already high cardiac tolerance to acute ischemia in neonatal rats; the first signs of protection were observed only at the beginning of the second postnatal week [3–5]. From these protective interventions, IPoC is the most promising for clinical use [6]. Our previous study failed to reveal any protective effect of IPoC protocols 3 × 10, 3 × 30, and 3 × 60 s during the first 10 days of postnatal development [5]. This finding slightly differs from our previous studies regarding IPC and adaptation to chronic hypoxia, where the protective effect was observed in 10-day-old animals. IPoC has multiple protocols and even in adult hearts, not all of them have protective effect in various species [7]. In order to find out the optimal cardioprotective protocol, we shortened IPoC intervals and increased their length (protocol 5 × 10 s) in this study.

The precise mechanisms of “conditioning” in the adult myocardium are still unclear and the same is also valid for the immature heart. In adult heart, they almost certainly involve the initial activation of “endogenous cardiac protective pathways” which probably include mitochondrial permeability transition pore (mPTP) [8], mitochondrial K-ATP channels (mito-K-ATP) [9], nitric oxide (NO) [10], reactive oxygen species (ROS) [11], and various protective kinases [12]. Blockade of either of these abolishes the positive effect of cardioprotective interventions in adult hearts [13]. The questions arise whether the same mechanisms are involved in the protection of the adult and immature heart and whether these mechanisms are also responsible for already high resistance of neonatal hearts.

It has been observed that mitochondria from neonatal heart are more resistant to mPTP opening [14]; however, they do not differ in the amount of the key mPTP regulatory protein, Cyclophilin D (Cyp-D), from adult hearts [15]. The role of other proposed mPTP components in ischemia–reperfusion (I/R) injury is still unclear even in adult hearts [16–18]. 5-hydroxydecanoate (5-HD), a blocker of mito-K-ATP channels, abolished protective effect of ischemic preconditioning in 7-day-old rabbits [19]. Nevertheless, in neonatal rats, 5-HD administered before ischemia had no effect on high neonatal resistance to I/R injury or IPC [4]. The same study, however, reported that the high resistance of neonatal hearts was abolished by eNOS blocker (L-NAME). Thus, L-NAME is the only substance known to decrease the high resistance of neonatal heart; however, data about the endogenous NO production in neonatal heart are not available.

For the explanation of discrepancy in the effect of L-NAME and 5-HD, short biological half-life of 5-HD [20, 21] should be taken into consideration; its application before ischemia may not have been sufficient to affect reperfusion. 5-min delay between applications of 5-HD and IPC was sufficient to significantly reduce its effect. The aim of our study was, therefore, firstly to evaluate the effect of 5-HD applied before and after ischemia (to eliminate possible effect of its short half-life) on high resistance of neonatal hearts to I/R injury and on the protective effect of IPoC.

Reactive oxygen species appear to be an important part of cardioprotective mechanisms in adult hearts (for review see [22]) and have been linked to the mito-K-ATP channel [23]. 3-NT is created by interaction of tyrosine with peroxynitrite, which is formed from NO and superoxide. Therefore, 3-NT levels should be related to both mito-K-ATP channel and NO production. The third aim was to measure the levels of nitrates and 3-nitrotyrosine (3-NT) as markers of endogenous NO production in neonatal animals.

## Methods

All investigations conform to the “Guide for the Care and the Use of Laboratory Animals,” published by the US National Institutes of Health. All procedures were approved by Animal Studies Committee of the Second Faculty of Medicine, Charles University. This article does not contain any studies with human participants performed by any of the authors.

### Animal model

A total of 142 neonatal Wistar rats age 1, 4, 7, and 10 days of both sexes were used throughout the experiments. Experimental and control groups were composed from at least three different litters. All mothers had free access to water and a standard laboratory diet *ad libitum*.

### Heart function

The animals were weighted, then killed by cervical dislocation. The chest was quickly opened and stainless steel cannula (with an external diameter of 0.45 mm for day 1 and 4 or 0.8 mm for day 7 and 10) was inserted into the ascending aorta. The heart was rapidly excised; the atria were removed and were perfused in the Langendorff mode under constant pressure corresponding to the mean arterial blood pressure for the given developmental stage [24, 25] i.e., 25, 42, 57, and 73 cm H_2_O on d 1, 4, 7, and 10, respectively. The hearts were perfused with a Krebs-Henseleit solution containing (in mmol/l): NaCl 118.0; KCl 4.7; CaCl_2_ 1.25; MgSO_4_ 1.2; NaHCO_3_ 25.0; KH_2_PO_4_ 1.2; glucose 7.0; and mannitol 1.1. The solution was saturated by a mixture of 95% O_2_ and 5% of CO_2_ (pH 7.4), and temperature was maintained at 37 °C. The hearts were electrically stimulated at a rate of 200 beats/min using silver electrodes attached to the base of the heart. The stimulation was performed with pulses of alternating polarity, 1-ms duration, and voltage set at 50% above the threshold level. The resting force was gradually increased by means of micromanipulator to the level at which the developed force (DF) was approximately 80% of the maximum force reached at the optimum preload. The contractile function of this isolated heart was measured using an isometric force transducer connected by a glass fiber, two-arm titanium lever, and silk suture (0.7 metric) to the apex of the heart. The DF (g) was evaluated automatically from the force signal using an online computer according to [3].

### Experimental protocol for ischemic postconditioning

**Fig. 1:**
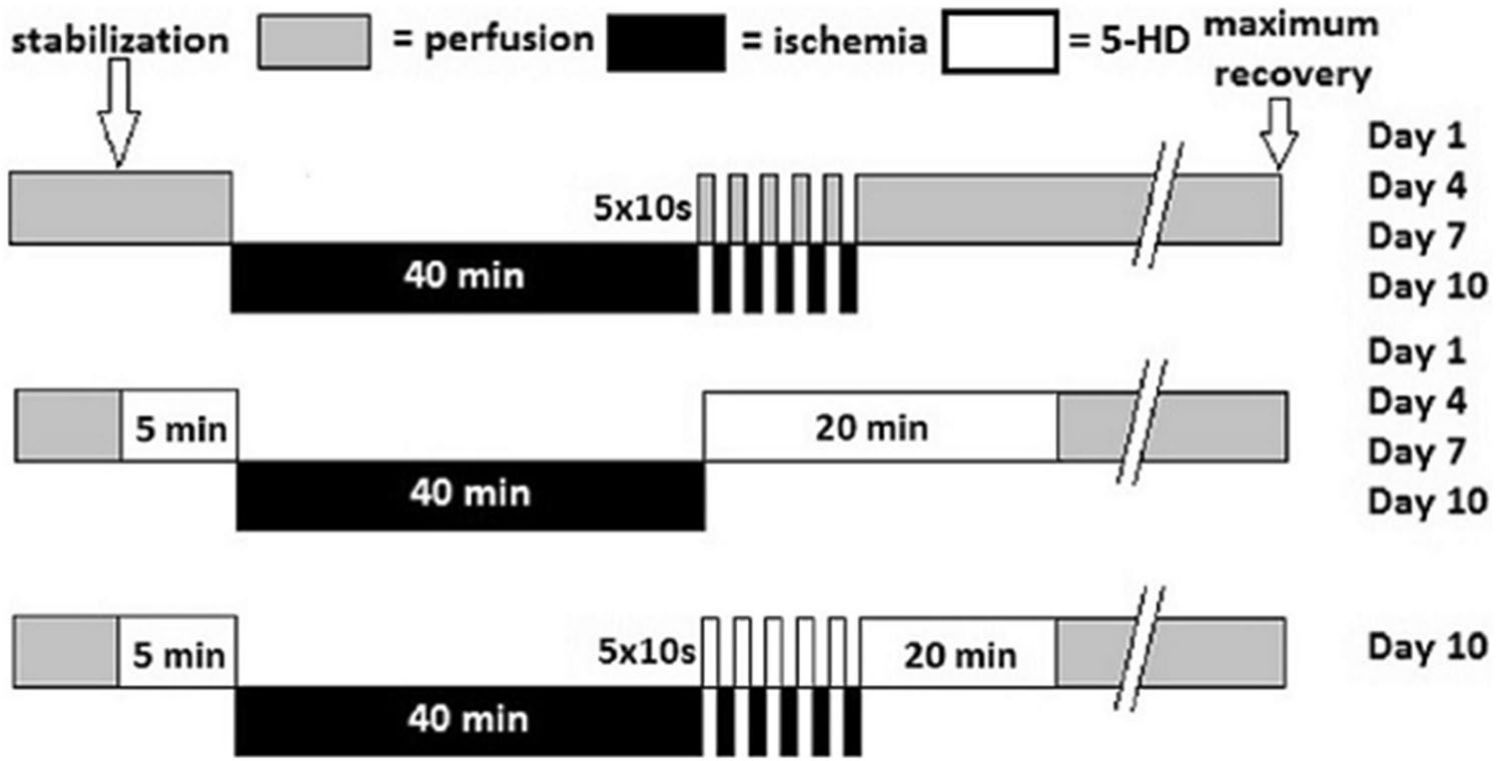
Scheme summarizing experimental groups and protocol of ischemic postconditioning

After a period of stabilization, baseline values of DF were recorded. The hearts from experimental group were exposed to 40 min of global ischemia. At the beginning of reperfusion, one-half of the hearts were postconditioned by subjecting them to five 10-s periods of global ischemia, each separated by the period of reperfusion of the same duration (Fig. 1). The remaining hearts were simply reperfused up to the maximum recovery of DF (the last value of DF before its decay). DF was measured in all hearts in 3-min intervals during the reperfusion period. Tolerance to ischemia was expressed as a recovery of DF (percentage of baseline values). After the experiment the heart weight was recorded.

### Experimental protocol for mito-K-ATP channel blockade

After a period of stabilization, baseline values of DF were recorded. One-half of hearts was perfused for 5 min with mito-K-ATP channel blocker, 5-hydroxydecanoate (Santa Cruz Biotechnology, Inc.) in Krebs-Henseleit solution at 500 µM concentration (the highest concentration found in other 5-HD studies [26]), control group was perfused with Krebs-Henseleit only. At the beginning of reperfusion, experimental hearts were perfused with identical 5-HD solution for another 20 min. Another experimental group of hearts was subjected to IPoC and 5-HD perfusion simultaneously. Control groups were perfused with Krebs-Henseleit solution only. All hearts were then reperfused with Krebs-Henseleit up to the maximum recovery of DF (the last value of DF before its decay). DF was measured in all hearts in 3-min intervals during the reperfusion period. Tolerance to ischemia was expressed as the recovery of DF (percentage of baseline values). After the experiment, the hearts were weighed.

### Isolation of hearts for NO analysis

The animals were weighted and killed by cervical dislocation and blood samples were taken. To obtain serum, the blood was centrifuged at 2900 rpm for 10 min, serum sample was then transferred into eppendorffs and frozen in liquid nitrogen until further analysis. The chest was quickly opened and heart was rapidly excised, atria were removed, the heart was weighted and washed with buffer (PBS—Phosphate Buffered Saline). In 1-day-old animals whole heart was frozen in liquid nitrogen until further analysis. In 10-day-old animals, right ventricle was removed and left ventricle with septum was frozen in liquid nitrogen until further analysis.

### Nitrates measurement

Serum sample (25 µl) was added to 5 ml of 0,1M VCl_3_ in 2M HCl with 300 µl of antifoaming agent. The sample was under constant bubbling with He and was heated to 90 °C. Nitrate, nitrite, and S-nitrosocompounds were converted to NO. To prevent damage to NO analyser (NOA), released gases were brought into cooler and gas bubbler filled with KOH to prevent damage from HCl vapor. In NOA (Sievers NOA 280i), NO reacts with O_3_ to form excited NO_2_^*^. As unstable electrons of NO_2_^*^ return to their original ground state, they dissipate energy. Light emission is linearly related to the NO content of the sample.

### 3-nitrotyrosine measurement

After pulverization and extraction, samples of heart tissue of 1-day and 10-day-old animals were used for estimation of 3-NT by competitive ELISA. The details of ELISA using our own monoclonal antibody against 3-NT and commercial anti-mouse IG rabbit antibody conjugated with peroxidase (Sigma A-8924) were described elsewhere [27]. 3-NT concentration was expressed per gram of extracted protein, determined by the bicinchoninic acid method [28].

### Statistical analysis

The results are expressed as means ± S.E.M. Each observation of IPoC was obtained from at least eight heart preparations in each group. Each observation of the effect of 5-HD was obtained from at least six heart preparations in each group. Body weight, heart weight, baseline, and recovery of DF values were evaluated by one-way ANOVA using Student-Newman–Keuls test. The data for IPoC and effect of 5-HD were also evaluated by one-way ANOVA using Student-Newman–Keuls test. The results of serum nitrates and 3-NT measurement were evaluated by unpaired t-tests. The statistical analyses were performed using StatView 5.0 (SAS Institute, Cary, N.C., USA). The figures were created using GraphPad Prism 6.07 for Windows (Graph Pad Software, La Jolla California USA). The results were considered statistically significant when *p* < 0.05.

## Results

### Tolerance to ischemia and effect of ischemic postconditioning

**Fig. 2:**
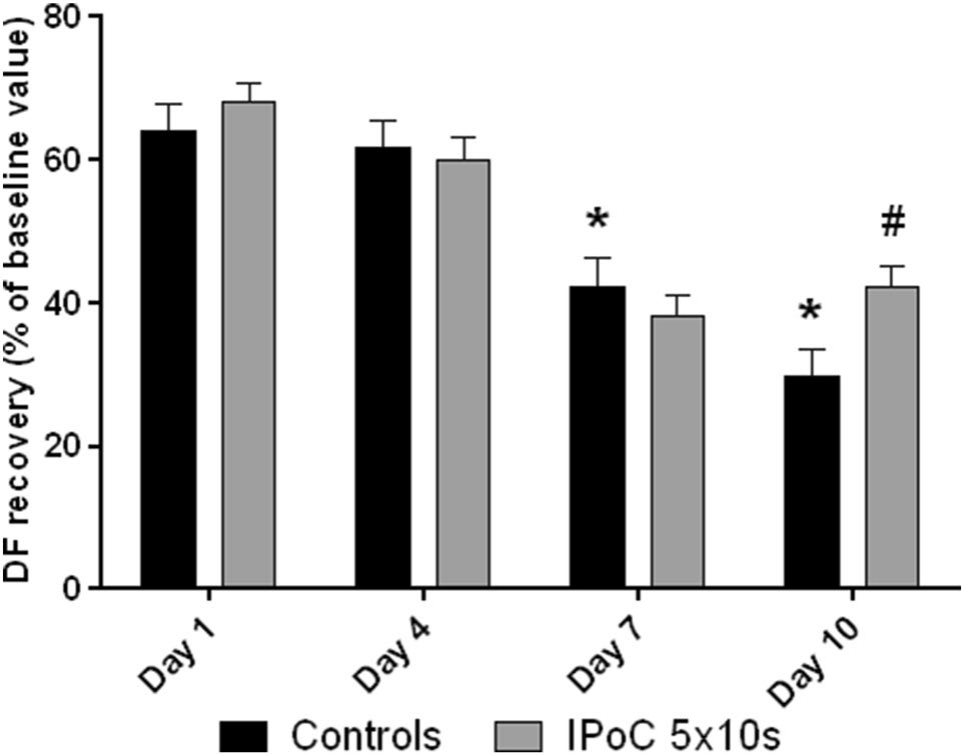
Tolerance to ischemia (DF, expressed as the percentage of baseline values) and the effect of ischemic postconditioning during postnatal development. *significantly different (*p* < 0.05) as compared to 1-day-old controls. **#**significantly different (*p* < 0.05) as compared to 10-day-old controls

Body and heart weights and baseline contractile parameters are summarized in Table 1. The body and heart weights as well as DF increased throughout the whole investigated period. Tolerance to ischemia, expressed as the postischemic recovery of DF, changed significantly during the first ten days of postnatal life. There was no significant difference between 1-day-old and 4-day-old animals; tolerance to ischemia then significantly declined on days 7 and 10 (Fig. 2). Based on the review by Skyschally et al. [7] and our previous study [5], a different protocol of ischemic postconditioning (5 × 10 s) was tested. This protocol significantly improved the recovery of DF on day 10, with no effect on days 1, 4, and 7 (Fig. 2).

**Table 1:** Morphometry and cardiac function parameters in 1, 4, 7, and 10 days old animals

Day	Protocol	n	Body weight (g)	Heart weight (mg)	HW/BW (mg/g)	DF (g)	Df/dt max (g/min)
1	Controls	12	6.67 ± 0.23	34.22 ± 1.33	5.15 ± 0.15	1.45 ± 0.11	35.25 ± 2.50
	5-HD	15	6.78 ± 0.22	33.05 ± 1.82	4.84 ± 0.16	1.40 ± 0.12	37.51 ± 4.47
	IPoC 5 × 10 s	9	6.07 ± 0.30	24.96 ± 0.70	4.10 ± 0.08	1.29 ± 0.07	32.63 ± 1.24
	Nitrates/3-NT	10	6.08 ± 0.28	26.40 ± 1.91	4.31 ± 0.14	-	-
4	Controls	9	9.16 ± 0.46	42.92 ± 2.01	4.73 ± 0.20	2.08 ± 0.13	48.48 ± 2.01
	5-HD	8	8.98 ± 0.56	45.76 ± 2.85	5.14 ± 0.23	2.28 ± 0.21	58.85 ± 5.37
	IPoC 5 × 10 s	8	10.55 ± 0.28	54.11 ± 3.20	5.15 ± 0.18	1.97 ± 0.15	51.92 ± 3.43
7	Controls	8	15.50 ± 0.81	72.26 ± 2.81	4.70 ± 0.10	3.08 ± 0.22	78.96 ± 4.70
	5-HD	6	17.77 ± 0.58	81.12 ± 2.15	4.58 ± 0.16	2.99 ± 0.29	71.44 ± 4.28
	IPoC 5 × 10 s	8	15.16 ± 0.36	71.35 ± 2.91	4.72 ± 0.21	2.99 ± 0.16	74.63 ± 3.41
10	Controls	8	20.01 ± 1.04	98.55 ± 4.02	4.99 ± 0.31	3.54 ± 0.23	80.96 ± 7.62
	5-HD	12	20.28 ± 0.64	92.76 ± 2.98	4.62 ± 0.19	3.59 ± 0.24	83.44 ± 5.41
	IPoC 5 × 10 s	10	18.81 ± 0.50	93.3 ± 2.53	4.97 ± 0.13	4.43 ± 0.30	100.30 ± 8.68
	IPoC 5 × 10 s + 5-HD	9	18.92 ± 0.37	86.77 ± 2.08	4.56 ± 0.13	5.22 ± 0.26	129.52 ± 5.46

*HW/BW* heart weight-to-body weight ratio, *DF* developed force, *Df/dt max* the most force rising ratio, *SEM* standard error of mean

### Effect of 5-hydroxydecanoate on control neonatal hearts

**Fig. 3:**
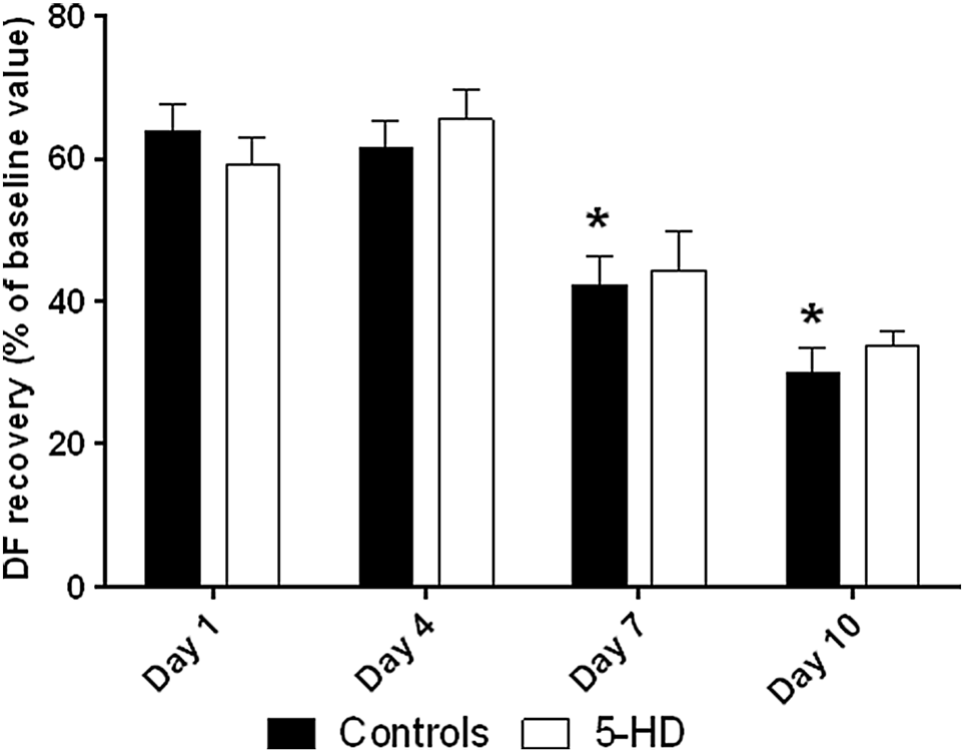
Tolerance to ischemia (DF, expressed as the percentage of baseline values) and 5-HD perfusion during postnatal development. *significantly different (*p* < 0.05) as compared to 1-day-old controls

**Fig. 4:**
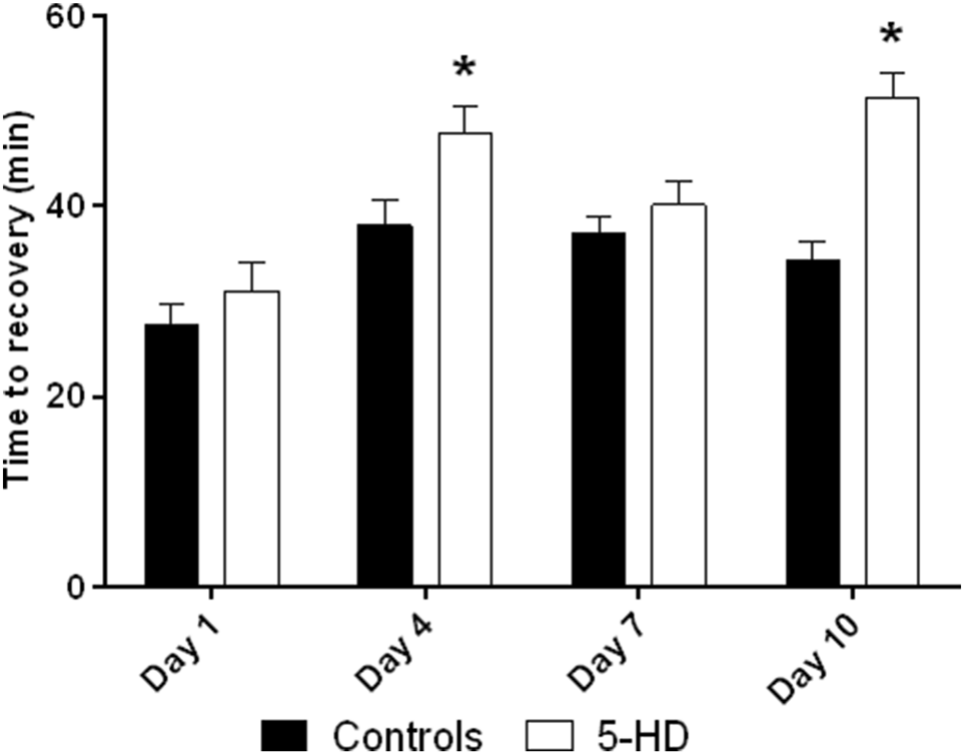
Time to recovery (min) in 5-HD perfusion. *significantly different (*p* < 0.05) as compared to correspondent 40-min ischemia group

Perfusion with 5-HD did not affect DF recovery on any postnatal day (Fig. 3). However, perfusion with 5-HD significantly affected time to (maximum) recovery on days 4 and 10 (Fig. 4, ontogenetic differences in control groups are in agreement with previous studies).

### Effect of 5-hydroxydecanoate on ischemic postconditioning

**Fig. 5:**
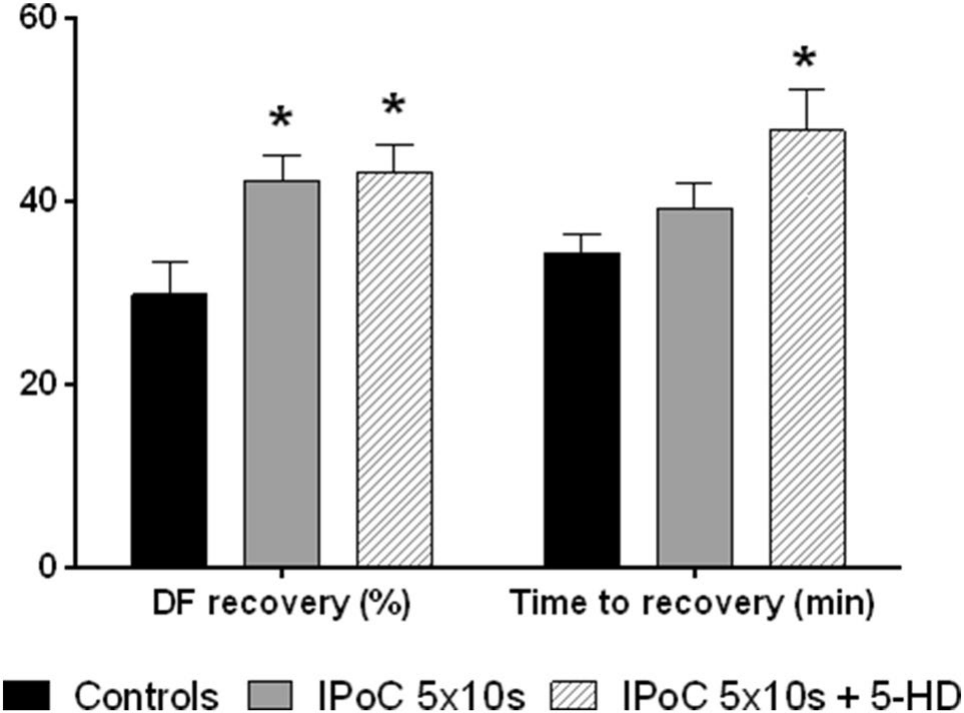
Tolerance to ischemia (DF, expressed as the percentage of baseline values) and time to recovery (min) in postconditioning and postconditioning with 5-HD in 10-day-old animals. *significantly different (*p* < 0.05) as compared to 40-min ischemia group

In adult hearts, IPoC is mito-K-ATP dependent [29]. We have observed that 5-HD did not affect the IPoC in neonatal heart. Time to recovery was significantly increased as in group perfused with 5-HD without IPoC (Fig. 5).

### Serum nitrate levels

**Fig. 6:**
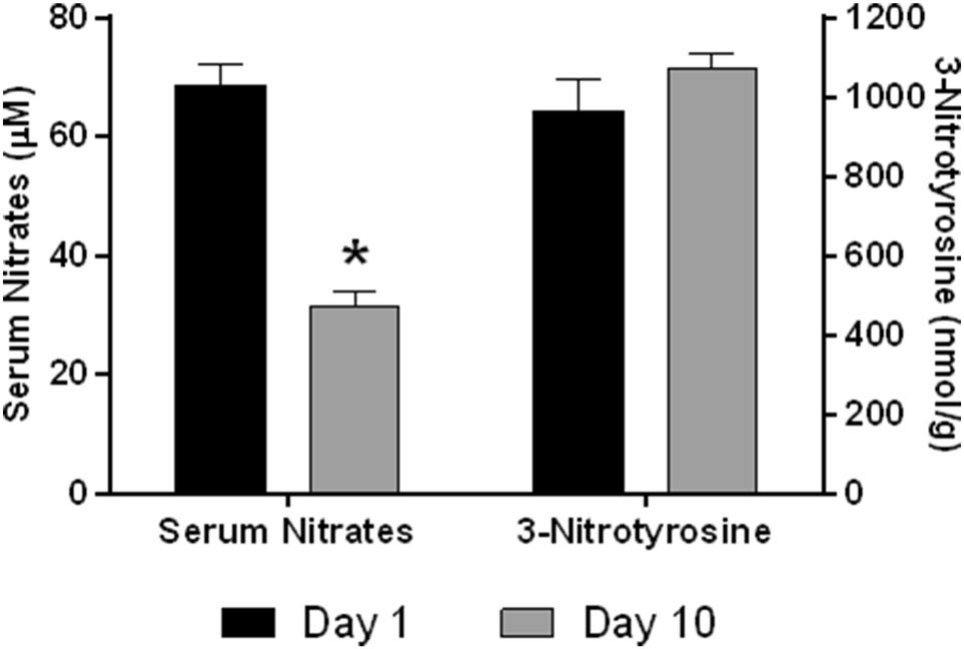
Serum nitrates levels and 3-nitrotyrosine in neonatal heart in 1-day-old and 10-day-old animals. *significantly different (*p* < 0.05) as compared to 1-day-old animals

In order to determine the possible role of NO in high tolerance of neonatal hearts to I/R injury, serum nitrate levels were measured. Our results showed significant decrease of serum nitrates levels between day 1 and day 10 (Fig. 6).

### 3-nitrotyrosine in neonatal heart

We found no difference in 3-NT content in neonatal hearts between day 1 and day 10 (Fig. 6).

## Discussion

Our current findings of IPoC development are in agreement with our previous studies with the protective effect of IPC and chronic hypoxia [3, 4] but slightly differ from our previous IPoC study [5], which failed to find the protective effect even on day 10 of postnatal life. Probable explanation is that protocol using 5 × 10 s is more effective than IPoC protocols (3 × 10 s, 3 × 30 s and 3 × 60 s) used in our previous study [5]. The data concerning the development of cardioprotective effect of IPoC would suggest the possible interference of IPoC mechanisms with the high resistance of neonatal hearts.

One of the cardioprotective mechanisms involved in the effect of IPoC in adult hearts is mito-K-ATP channel. However, 5-HD in our study failed to affect both the resistance as well as the cardioprotective effect of IPoC in neonatal hearts; it seems, therefore, that neither of them are induced by mito-K-ATP channel opening. Because the structure of mito-K-ATP channel is not well known even in adult heart [30], one possible explanation may be that 5-HD does not affect neonatal mito-K-ATP at all. If 5-HD actually affects mito-K-ATP of the neonatal heart, then it would implicate difference in the mechanism of cardioprotective effect of IPoC between neonatal and the adult hearts. Moreover, interspecies differences may be responsible for discrepancies between the rat and rabbit studies (compare [4] with [19]). Unexpected result is the effect of 5-HD on the time to recovery, which could be probably explained as the effect on contractility known from adult hearts [31] rather than effect on I/R injury. Time to (maximum) recovery is not related to cardiac tolerance to ischemia (compare Figs. 3, 4).

The increased serum nitrate levels in 1-day-old rats thus suggest increased NO production in neonatal hearts as one possible cause of its high resistance. The NO has cardioprotective effect in adult hearts [32] and L-NAME reduced protective effect of IPoC [10]. L-NAME also abolished the high tolerance of neonatal heart to I/R injury [4]. However, source of this NO production in neonatal heart remains to be identified. In adult hearts, eNOS activated by Akt kinase was described as a very likely source of NO production [33]. Although neonatal hearts have larger pool of Akt kinase than adult hearts, it does not appear to be constitutively active [34]. How NO exerts its cardioprotective effect in neonatal heart remains to be clarified as well; in adult hearts S-nitrosylation has been proposed [35]. The fact that no similar difference was found in 3-NT could be possibly explained as a lack of reactive oxygen species (superoxide) needed for peroxynitrite formation, confirming that ROS and mito-K-ATP probably do not play role in the resistance of neonatal heart to I/R injury.

Our study in the early postnatal period tested two mechanisms, which play the role in cardioprotection of adult hearts. But there are some other possibilities. Historically, mechanisms such as glycolytic capabilities, calcium metabolism, or vascularization were suggested to be responsible for high neonatal tolerance to ischemia (for a review see [36]). Furthermore, it is necessary to mention the role of mitochondrial mPTP pore, which is commonly referred as a key structure in mechanisms of cardioprotection in adult hearts. It has been observed that mitochondria from neonatal hearts are more resistant to calcium-induced mPTP opening [14]; however, this is not induced by differences in the amount of Cyp-D, one of the structural compounds of mPTP pore [15]. Recently, differences in protective kinases glycogen synthase kinase 3β and Akt in early ontogenetic development were described [34]. The role of pH and other signaling pathways, known from the adult myocardium (for a review see [37]), were not described in neonates yet. On the contrary, differences in myocardial sarcoplasmic calcium dependence between adult (where calcium plays significant role in cardioprotection [38]) and neonatal hearts are well known. However, timecourse of resistance to I/R injury in neonatal heart and sensitivity of neonatal heart to calcium blockers differs (compare [39] with [3]). Thus, this ontogenetic difference is unlikely to be responsible for high tolerance of neonatal heart to ischemia.

To fully understand the mechanisms of the high tolerance to ischemia and cardioprotective mechanisms of neonatal heart, detailed knowledge of early postnatal development is essential. The developing heart undergoes many changes and not all of them need to be necessarily responsible for its high resistance to I/R injury. In conclusion, the resistance of 1-day-old rats to I/R injury seems to reach a maximum that cannot be surpassed, no matter what cardioprotective interventions were applied. Increased NO production appears to be a key factor in this resistance.
